# The Impact of Body Mass Index on the Duration of Hospital Stay After Cardiac Surgery

**DOI:** 10.7759/cureus.84985

**Published:** 2025-05-28

**Authors:** Zahraa Saker, Mohamad Saab, Ali Rabah

**Affiliations:** 1 Department of Clinical Research, Al-Rassoul Al-Aazam Hospital, Beirut, LBN; 2 Department of Cardiovascular and Thoracic Surgery, Beirut Cardiac Institute, Beirut, LBN; 3 Division of Electrophysiology, Beirut Cardiac Institute, Beirut, LBN

**Keywords:** body mass index (bmi), cardiac surgery, coronary artery bypass graft procedure (cabg), isolated valve surgery, length of hospital stay

## Abstract

Background: The impact of body mass index (BMI) on clinical outcomes after cardiac surgery remains controversial. The objective of this study was to evaluate the association between BMI and the duration of hospital stay in patients after cardiac surgery.

Method: A retrospective, cross-sectional study was conducted with 1120 patients admitted to Beirut Cardiac Institute for cardiac surgery between July 2020 and July 2022. Patients were divided based on the BMI groups as follows: normal weight, overweight, obesity class I, obesity class II, and obesity class III. The length of hospital stay after cardiac surgery was compared among BMI groups after cardiac surgery, and also among each type of surgery.

Results: Of the 1120 patients who underwent cardiac surgery, 270 (24.1%) patients had normal BMI, 459 (41.0%) patients were overweight, and 391 (34.9%) patients were obese. The average length of hospital stay was 8.97 ± 5.88 days, with no correlation between BMI and length of hospital stay (r = 0.058; P = 0.052). Obesity class III patients experienced the longest stay (11.37 ± 7.09 days) with a significant difference compared to the length of hospital stay of normal weight patients (P = 0.007). Patients who underwent coronary artery bypass graft (CABG) surgery experienced the shortest duration of stay (8.50 ± 4.81 days) compared to the isolated valve surgery (9.46 ± 8.12 days) and combined CABG and valve surgery (10.52 ± 4.56 days; P ≤ 0.001). Within the CABG group, obesity class III patients had experienced the longest length of hospital stay (13.05 ± 8.85 days) with a significant difference compared to the length of hospital stay in normal weight patients (7.93 ± 3.11 days; P = 0.004).

Conclusion: Our results demonstrated no correlation between BMI and length of hospital stay after cardiac surgery. However, the obesity class III patients within the CABG group had the longest length of hospital stay. These findings highlight the importance of shifting the focus of healthcare planning to obesity class III, particularly in the context of specific surgical procedures.

## Introduction

Cardiac surgery is a complex and high-risk procedure that requires intensive critical care during the postoperative period. One of the risk factors that influences clinical outcomes following cardiac surgery is the body mass index (BMI), which is widely used as a standard indicator of body fat status in clinical and epidemiological research [1,2].

The impact of BMI on the postoperative outcomes of cardiac surgery is multifaceted and complex. Previous studies have suggested an association between high BMI and an increased risk of complications following cardiac surgery, such as prolonged ventilation duration, atrial fibrillation, increased duration of hospitalization, and death [1,3,4]. However, conflicting findings were reported by other studies. While Johnson et al. reported lower perioperative adverse outcomes and mortality among overweight and obese patients compared to normal BMI patients [5], Parlow et al. observed longer postoperative ventilation for normal BMI and overweight patients [6]. Furthermore, Zhang et al. stated a longer survival rate for overweight patients [7]. Interestingly, the specific impact of BMI on the length of hospital stay after cardiac surgery remains relatively debatable. The aim of the current study is to investigate the impact of BMI on the duration of hospital stay and evaluate the obesity debate following cardiac surgery.

## Materials and methods

A retrospective, cross-sectional study was conducted using the archived medical database of cardiac surgeries in the Division of Cardiothoracic and Vascular Surgery at Beirut Cardiac Institute (BCI), Beirut, Lebanon, from July 2020 to July 2022. Demographic, medical, and nutritional information of the patients who underwent cardiac surgeries was collected and entered retrospectively in a prespecified database by dedicated data coordinating personnel. The present study was approved by the Institutional Review Board (IRB) of BCI. Patients’ confidentiality was maintained at all times.

The inclusion criteria were all patients, 18 years and older, who underwent coronary artery bypass graft (CABG) surgery, valve repair/replacement, or combined CABG and valve repair/replacement. Exclusion criteria were patients younger than 18 years, and other cardiac surgeries such as pericardiectomy, pericardial window, coarctation of the aorta, auricle closure, Bentall surgery, ascending aorta replacement, atrial septal defect closure, and myxoma excision. The nutritional status of the patients was established based on their BMI. BMI was classified according to the World Health Organization (WHO) for data analysis as follows: underweight (BMI < 18.5 kg/m^2^), normal (18.5 ≤ BMI < 25 kg/m^2^), overweight (25 ≤ BMI < 30 kg/m^2^), obesity class I (30 ≤ BMI < 35 kg/m^2^), obesity class II (35 ≤ BMI < 40 kg/m^2^), and obesity class III (BMI ≥ 40 kg/m^2^) [2]. Underweight patients (n = 11) were excluded from the study to ensure accurate comparison.

Numerical variables were described using means and standard error of deviations (SED), while categorical variables were represented in frequencies and percentages. Statistical analysis was conducted using the IBM SPSS statistical software package version 26.0 (IBM Corp., Armonk, NY). Mann-Whitney U test and Kruskal-Wallis test were performed to compare continuous variables, whereas the chi-square test was used to compare categorical variables. The simple linear regression model was applied to test the correlation between BMI values and length of hospital stay. A P-value < 0.05 was considered statistically significant in this study.

## Results

A total of 1120 patients hospitalized in BCI for cardiac surgery from July 2020 to July 2022 met the inclusion criteria and were included in the study. As presented in Table 1, the mean age of the patients was 63.78 ± 11.14 years, and 311 (27.8%) were older than 70 years. The majority of the patients were males (776, 69.3%). The average weight was 77.56 ± 14.94 kg, and the mean BMI was 28.74 ± 5.13 kg/m^2^. Among 1120 patients, 270 (24.1%) had a normal BMI, 459 (41.0%) were overweight, and 391 (34.9%) were obese. CABG was performed in 706 (59.9%) of patients, representing the highest frequency of performed cardiac surgery.

**Table 1:** Clinical characteristics of the patients. Values are represented by n (%) or means ± standard error of deviations. BMI: body mass index; CABG: coronary artery bypass graft; CAD: coronary artery disease; CKD: chronic kidney disease; COPD: chronic obstructive pulmonary disease; EF: ejection fraction.

Characteristic	N = 1120 (%)
Age (year)	63.78 ± 11.14
Gender	
Male	776 (69.3)
Female	344 (30.7)
Weight (kg)	77.56 ± 14.94
BMI (kg/m^2^)	28.74 ± 5.13
Normal	270 (24.1)
Overweight	459 (41.0)
Obese	391 (34.9)
EF ≤ 40%	162 (14.5)
Smoking	722 (64.5)
Hypertension	870 (77.7)
Diabetes	504 (45.0)
Dyslipidemia	545 (48.7)
CAD	798 (71.3)
COPD	318 (28.4)
CKD	189 (16.9)
Surgery	
CABG	706 (59.9)
Valve	295 (25.0)
CABG & valve	119 (10.1)

Demographic characteristics and preoperative risk profiles of all BMI groups are represented in Table 2. Overweight patients were less likely to have chronic kidney disease (218, 47.5%) and more likely to undergo CABG surgery (302, 65.8%) and isolated valve surgery (114, 24.8%) compared with the normal BMI patients. On the other hand, obese patients were more likely to be females (156, 39.9%), and have hypertension (320, 81.8%), dyslipidemia (207, 52.9%), and undergo isolated valve surgery (127, 32.5%); yet less likely to be smokers (237, 60.6%) and have reduced ejection fraction (38, 9.7%) compared to the normal BMI patients.

**Table 2:** Patients’ characteristics in normal BMI, overweight, and obese groups. * A p-value of less than 0.05 was considered significant. CABG: coronary artery bypass graft; CAD: coronary artery disease; CKD: chronic kidney disease; COPD: chronic obstructive pulmonary disease; EF: ejection fraction; P1: P-value of overweight patients compared with normal BMI patients; P2: P-value of obese patients compared with normal BMI patients.

Characteristic	Normal BMI (n = 270)	Overweight (n = 459)	P_1_ value	Obese (n = 391)	P_2_ value
Age (year)	63.97 ± 11.99	63.65 ± 10.68	0.531	63.80 ± 11.10	0.698
Gender			0.417		<0.001*
Male	205 (75.9)	336 (73.2)		235 (60.1)	
Female	65 (24.1)	123 (26.8)		156 (39.9)	
Weight (kg)	63.95 ± 9.59	75.48 ± 9.06	<0.001*	89.41 ± 14.36	<0.001*
EF ≤ 40%	54 (20.0)	70 (15.3)	0.115	38 (9.7)	<0.001*
Smoking	187 (69.3)	298 (64.9)	0.264	237 (60.6)	0.023*
Hypertension	204 (75.6)	346 (75.4)	0.958	320 (81.8)	0.050*
Diabetes	115 (42.6)	202 (44.0)	0.71	187 (47.8)	0.184
Dyslipidemia	120 (44.4)	218 (47.5)	0.425	207 (52.9)	0.032*
CAD	191 (70.7)	321 (69.9)	0.818	286 (73.1)	0.498
COPD	85 (31.5)	121 (26.4)	0.138	112 (28.6)	0.433
CKD	57 (21.1)	69 (15.0)	0.036*	63 (16.1)	0.101
Surgery			0.018*		<0.001*
CABG	173 (64.1)	302 (65.8)		231 (59.1)	
Valve	54 (20.0)	114 (24.8)		127 (32.5)	
CABG & valve	43 (15.9)	43 (9.4)		33 (8.4)	

**Figure 1:**
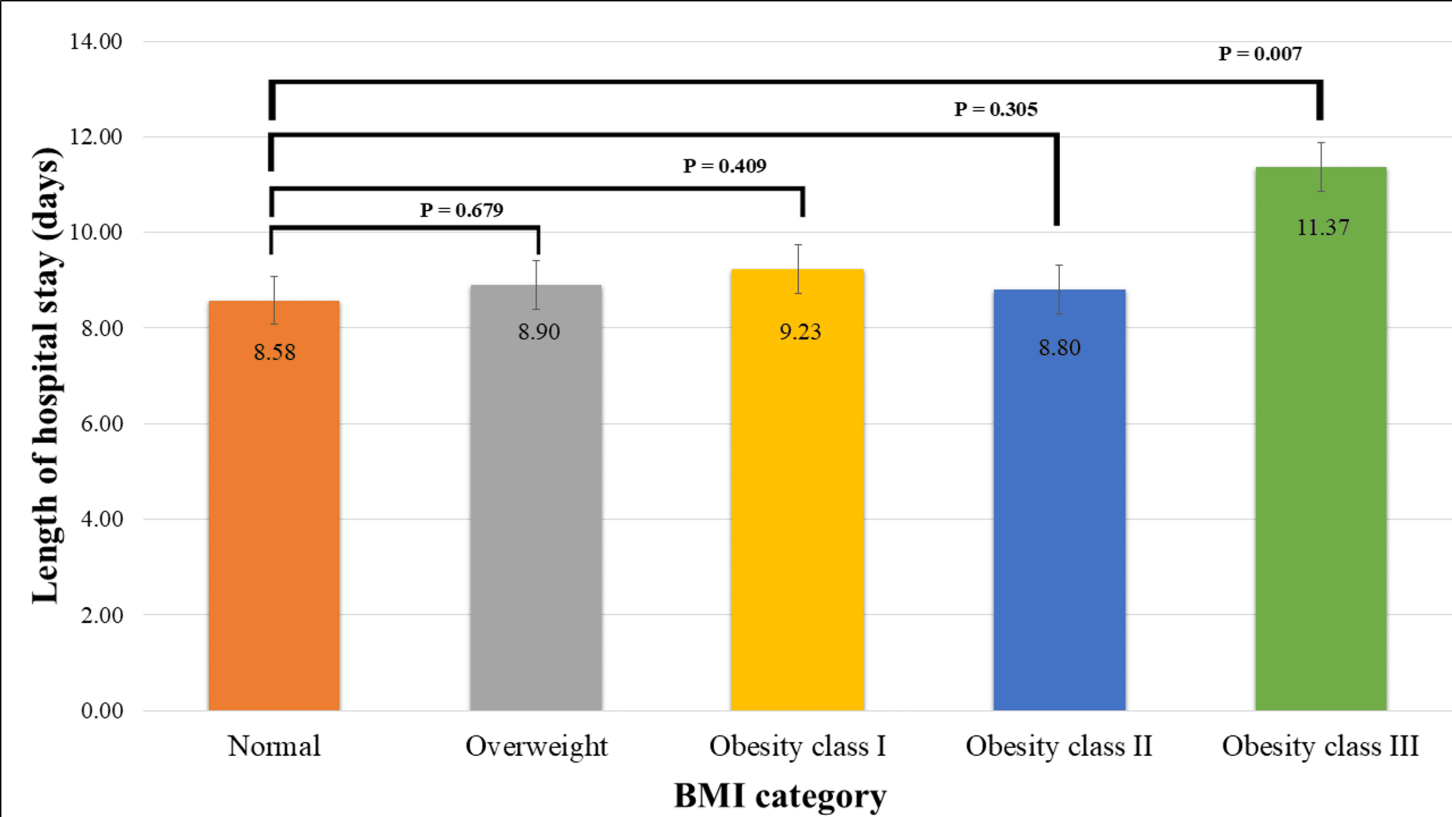
Length of hospital stay after cardiac surgery based on BMI groups.

**Figure 2:**
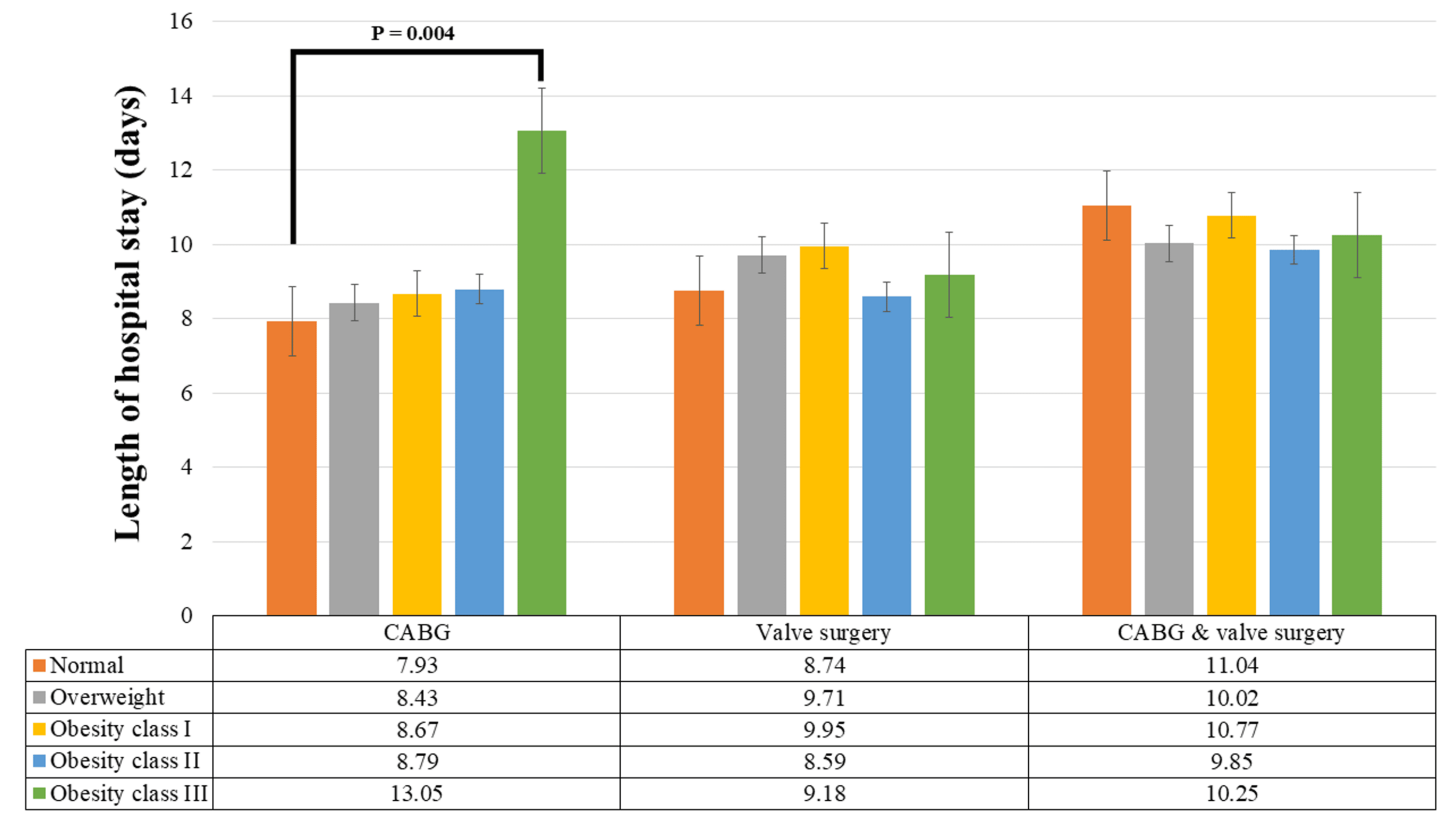
Length of hospital stay after cardiac surgery based on surgery type. CABG: coronary artery bypass graft.

The average length of hospital stay was 8.97 ± 5.88 days, with no correlation between BMI and length of hospital stay (r = 0.058; P = 0.052). Obesity class III patients experienced the longest stay (11.37 ± 7.09 days), with a significant difference compared to the length of hospital stay of normal BMI patients (8.52 ± 4.62 days) (P = 0.007) (Figure 1). Patients who underwent CABG surgery experienced the shortest duration of stay (8.50 ± 4.81 days) compared to the isolated valve surgery (9.46 ± 8.12 days) and combined CABG and valve surgery (10.52 ± 4.56 days) (P ≤ 0.001). Within the CABG group, obesity class III patients displayed the longest length of hospital stay (13.05 ± 8.85 days) with a significant difference compared to the length of hospital stay in normal BMI patients (7.93 ± 3.11 days) (P = 0.004) (Figure 2). The percentage increase in hospital stay for obesity class III patients compared to the normal BMI group was 64.56%. However, the length of hospital stay was not significantly associated with BMI group in patients who underwent valve surgery or combined CABG and valve surgery.

## Discussion

Obesity is an alarmingly widespread public health concern with a substantial effect on health outcomes, affecting, along with overweight, about a third of the world’s population today [8,9]. One area where obesity has a pronounced effect is the postoperative care following cardiac surgery [4]. A prolonged hospital stay after cardiac surgery usually leads to a higher incidence of complications, delayed recovery, as well as increased healthcare costs [10]. Understanding the association between BMI and the length of hospital stay following cardiac surgery is of great consequence for improving surgery outcomes and optimizing patient care strategies. Moreover, investigating the impact of BMI on the length of hospitalization may help healthcare providers better tailor management policies to meet the needs of patients based on their BMI values.

Although this single-center study showed no association between BMI and the length of hospital stay after cardiac surgery, obesity class III patients who underwent CABG were more likely to experience a longer length of stay compared to normal BMI patients. Numerous studies revealed similar results of prolonged length of stay in association with higher BMI after cardiac surgery [10-15]. However, Stamou et al. have reported a conflicting result of shorter hospital stays for overweight patients compared to normal BMI patients [16]. Yet, the absence of a correlation between BMI and postoperative length of hospital stay was stated in many studies [1,17-21].

Interestingly, and consistent with previous studies, this study demonstrated a significant difference in the length of stay between the surgery types, with the shortest duration of stay for the patients who underwent CABG compared to those who underwent isolated valve surgery and combined CABG and valve surgery [10,22-24]. These findings suggested that patients undergoing CABG may experience faster recovery and encounter fewer postoperative complications, leading to shorter hospital stays. As there are numerous factors implicated in prolonged hospital stays post cardiac surgeries [25], BMI and type of surgery were considered predictors of prolonged postoperative hospital stays [10].

Furthermore, as the shortest stay was experienced by patients who underwent CABG, the longest duration of hospital stay was reported for the obesity class III patients. Our findings were consistent with those of Habib and colleagues, who demonstrated significantly longer hospital stays for morbid obesity (obesity class III) post CABG [14]. In contrast, isolated valve surgery and combined CABG and valve surgery showed no significant differences regarding BMI groups and length of hospital stay within each surgery type. In line with our findings, Bruno et al. suggested no significant association between the length of hospital stay and BMI groups following valve surgery [21]. However, a large study of valve surgery showed that obesity class III patients experienced longer hospital stays compared to other BMI groups [26]. Although there is no definite or reasonable explanation for such contradictory results in our study and the recent research, these discrepant results raise a question about the impact of BMI on recovery time based on the type of surgery.

Our study had a well-defined research question focusing on the association between BMI and length of hospital stay following cardiac surgery, and also according to the type of surgery. The number of patients included was relatively large, which increased the statistical power and generalizability of the results in our population. In addition, our results may have clinical implications for managing obese patients undergoing CABG. On the other hand, our study had inherent limitations that introduced bias and restricted its generalizability, such as limited outcome measures like postoperative or long-term complications. Additionally, single-center studies may also reduce the generalizability of the obtained results to other healthcare centers with different populations and healthcare practices. Furthermore, the potential effect of some confounders, such as socioeconomic level and pre-existing comorbidities, could influence the relationship between BMI and length of hospital stay. These strengths and limitations should be considered when designing future research.

## Conclusions

In conclusion, our study highlights that obesity class III patients undergoing CABG tend to experience prolonged hospitalization periods. This suggests the potential impact of obesity on postoperative outcomes and the recovery process. The observed difference in the length of hospital stay between different cardiac surgery types based on BMI underscores the importance of considering the patient’s clinical characteristics, including BMI, upon planning for cardiac procedures. Further research involving more diverse surgical procedures and bigger samples is needed to validate the obtained results. Moreover, understanding the association between BMI and length of hospital stay may help healthcare providers better manage patients’ care strategies and improve the recovery process for patients undergoing cardiac surgery.
